# 3D brain Organoids derived from pluripotent stem cells: promising experimental models for brain development and neurodegenerative disorders

**DOI:** 10.1186/s12929-017-0362-8

**Published:** 2017-08-20

**Authors:** Chun-Ting Lee, Raphael M. Bendriem, Wells W. Wu, Rong-Fong Shen

**Affiliations:** 1 Facility for Biotechnology Resources, Center for Biologics Evaluation and Research, FDA, Silver Spring, MD 20993 USA; 2 Center for Neurogenetics, Feil Family Brain and Mind Research Institute, Weill Cornell Medicine, New York, NY 10021 USA; 3 Center for Biologics Evaluation and Research, U.S. Food and Drug Administration, Building 52, Rm 1121, 10903 New Hampshire Avenue, Silver Spring, MD 20993 USA

**Keywords:** 3D brain organoids, Induced pluripotent stem cells, Brain development, Neocortex, Neurodevelopmental disorder, Neurodegenerative disorder, Microcephaly, Drugs, Autism, Alzheimer’s disease

## Abstract

Three-dimensional (3D) brain organoids derived from human pluripotent stem cells (hPSCs), including embryonic stem cells (ESCs) and induced pluripotent stem cells (iPSCs), appear to recapitulate the brain’s 3D cytoarchitectural arrangement and provide new opportunities to explore disease pathogenesis in the human brain. Human iPSC (hiPSC) reprogramming methods, combined with 3D brain organoid tools, may allow patient-derived organoids to serve as a preclinical platform to bridge the translational gap between animal models and human clinical trials. Studies using patient-derived brain organoids have already revealed novel insights into molecular and genetic mechanisms of certain complex human neurological disorders such as microcephaly, autism, and Alzheimer’s disease. Furthermore, the combination of hiPSC technology and small-molecule high-throughput screening (HTS) facilitates the development of novel pharmacotherapeutic strategies, while transcriptome sequencing enables the transcriptional profiling of patient-derived brain organoids. Finally, the addition of CRISPR/Cas9 genome editing provides incredible potential for personalized cell replacement therapy with genetically corrected hiPSCs. This review describes the history and current state of 3D brain organoid differentiation strategies, a survey of applications of organoids towards studies of neurodevelopmental and neurodegenerative disorders, and the challenges associated with their use as in vitro models of neurological disorders.

## Background

In recent years, genomic studies of individuals with developmental or neurodegenerative diseases have tremendously advanced our understanding of the genetics of these disorders [1–5]. Many of them, including autism spectrum disorder (ASD), schizophrenia, Alzheimer’s disease (AD), and Parkinson’s disease (PD), are caused by a heterogeneous combination of variant alleles and have therefore proven difficult to recapitulate in animal models, which are better suited for studying single-gene mutations [6–9]. Although the human brain is the ideal model to study human neuropathology, the limited availability of healthy and diseased brain tissue as well as the difficulties with culturing or genetically manipulating the tissue makes this an inopportune model with which to easily elucidate molecular mechanisms underlying these disorders. Recent progress in iPSC technology has provided a remarkable alternative for the study of human brain diseases through the scalable, manipulatable production of human neural cells derived directly from patients with diverse neurological diseases [10–15]. However, the potential of hiPSCs to recapitulate complex human brain disorders remains incompletely exploited. In this review, we summarize the current state of using hPSC-derived in vitro 3D models of brain development and neurodegenerative disorders and discuss the advantages and challenges of employing 3D human brain models for drug screening and investigating mechanisms behind these neurological disorders.

## 2D hPSC neural differentiation models

2D neural tube-like rosettes, made up of neural progenitors organized radially around ventricle-like cavities, have previously been generated from hPSCs to study neuronal development [16–22]. Through specialized differentiation protocols, these 2D culture models have the ability to produce specified and highly homogeneous cell populations, such as excitatory and inhibitory cortical neurons [16, 17, 20], midbrain dopaminergic neurons [18, 19], and motor neurons [19, 21, 22]. Recently, Kindberg et al. were able to mimic neocortical development in 2D culture conditions to generate both glutamatergic and GABAergic cortical neurons on radial glial scaffolding by employing neocortical trophic factors FGF18, BDNF, and NT3 [23]. Notably, the glutamatergic projection neurons were generated in a temporally sensitive manner mimicking in vivo neocortical development, with deep-layer CTIP2^+^ neurons appearing first, followed by generation of upper-layer SATB2^+^ neurons. Moreover, Lee et al. have developed a 2D neural culture system for producing connections between neurons from two distinct brain regions, the neocortex and midbrain [24]. However, the inability of these 2D models to recapitulate in vivo-like cytoarchitectural organization and synaptic connections of the brain has impeded their use in precise disease modeling or drug screening applications.

## 3D mini-brains

### hPSC-derived 3D neural cell aggregates

A variety of protocols are available for differentiating hPSCs into 3D neural cell aggregates. The 3D human neural model developed by Hogberg et al. [25] employs similar approaches to those established for 2D rosette culture [16]. 3D neural cell aggregates can be generated from either single human neural progenitors or neural rosettes cultured in a 3D suspension, although more efficient neural induction has been observed in the single cell-derived aggregated cultures than in rosette-derived cultures. Kim et al. generated 3D neural cell aggregates instead by embedding immortalized human neural precursors in Matrigel containing high levels of neural-based extracellular matrix proteins [26, 27]. Furthermore, using genetically modified human neural precursors overexpressing mutant *APP* and *PSEN1*, their 3D model successfully mimicked β-amyloid (Aβ) accumulation, previously suggested to be associated with AD pathogenesis [28, 29]. Both Aβ deposition and Aβ-driven neurofibrillary tangles formation were observed in 3D aggregates but not in 2D cultures, possibly due to the more limited diffusion of Aβ in tightly-packed 3D aggregates than in 2D cultures [26, 27]. Although 3D neural aggregates provide the potential for neurodegenerative disease modeling, the lack of cytoarchitectural organization or cellular diversity in these 3D models will impede the recapitulation of complex brain development processes that are dependent on cell-cell interactions across different brain regions.

### hPSC-derived 3D brain organoids

Recently, complex 3D arrangements of cells resembling human brain tissue, termed brain organoids, have been generated from hPSCs (Table 1). The most widely used protocol for the generation of 3D brain tissue from hPSCs is the serum-free floating culture of embryoid body-like aggregates with quick reaggregation (SFEBq), a protocol that incorporates embryoid body (EB)-like formation by reaggregation of dissociated hPSCs in the presence of a Rho kinase inhibitor [30–32]. These EB-like aggregates cultured in suspension progressively differentiate into several polarized neuroepithelial rosettes structurally similar to the in vivo cortical neuroepithelium that subsequently generate layer-specific cortical neurons [32]. The cortical progenitors generated this way are heterogeneous with respect to their rostral-caudal identities. The rostral-caudal polarity of these cells can be manipulated by modulation of FGF signaling. The activation of FGF signaling promotes rostralization of SFEBq-induced cortical progenitors while the attenuation of FGF signaling promotes a caudal fate. Moreover, the SFEBq-induced progenitors can be directed towards caudal-dorsal pallial tissues, hem and choroid plexus, by WNT and BMP signals. Thus, specific regional character of SFEBq-induced tissues can be selectively modulated by physiologically relevant patterning factors. This method has been further optimized to promote more protracted cortical development by the addition of IWR1e (WNT inhibitor), SB431542 (TGFβ inhibitor), fetal bovine serum, heparin, and Matrigel, and by the extension of the culture period from 46 to 112 days [33]. The optimized culture produces human-specific outer radial glia (oRG) progenitor cells predominantly found in the human outer subventricular zone (oSVZ) of the neocortex. Furthermore, early-born neurons migrate to deep layers of the cortical plate of self-organized cortical tissues whereas later-born neurons migrate to upper layers, consistent with the in vivo inside-out patterning of the human neocortex. This improved SFEBq culture therefore potentially provides a useful model with which to explore the role of oRGs in human brain development.

**Table 1:** Human PSC-derived 3D brain organoid models

Brain region in organoid	Type of PSCs	Patterning factor	Extracellular Scaffolding	Spinning bioreactor	# of VZ-like regions in organoid	Days of differentiation	Reference
Rostral and caudal cortices, hem and choroid plexus	Human ESCs	Initial stage: Dkk-1, Lefty-1	-	-	Inconsistent multiple	46 days	[32]
		Rostral cortices: Fgf8					
		Caudal cortices: Fgf inhibitor FGFR3-Fc					
		Cortical hem and choroid: Wnt3a and BMP4					
Neocortex	Human ESCs	IWR1e, SB431542	-	-	Inconsistent multiple	112 days	[33]
Dorsal cortex, ventral forebrain, retina, hippocampus, choroid plexus, midbrain-hindbrain boundary	Human ESCs/iPSCs	-	Matrigel	Yes	Inconsistent multiple	75 days	[34]
Forebrain, midbrain, hypothalamus	Human iPSCs	Forebrain organoids: dorsomorphine, A83–01, WNT3A, CHIR99021, SB-431542	Matrigel	Yes	Inconsistent multiple	120 days	[36]
		Midbrain organoids: LDN-193189, SB-431542, SHH, purmorphamine, FGF-8, CHIR99021					
		Hypothalamus organoids: LDN-193189, SB-431542, 1-Thioglycerol, WNT3A, SHH, purmorphamine					
Cerebral cortex	Human iPSCs	Dorsomorphin, SB431542, bFGF, EGF	-	-	Inconsistent multiple	181 days	[37]
Neocortex	Human ESCs/iPSCs	SB431542, LDN193189, PD0325901, bFGF, FGF18	-	-	1	66 days	[40]

Lancaster et al. used a modified SFEBq method to yield a novel hPSC-based 3D brain model called cerebral organoids [34]. Also known as “mini-brains”, these cerebral organoids contain discrete brain regions including dorsal cortex, ventral forebrain, retina, hippocampus, choroid plexus, and midbrain-hindbrain boundary. To accomplish this, Lancaster et al. generated EBs from dissociated single hPSCs and once EBs had been neutrally induced to a neuroectodermal fate, they were embedded in Matrigel droplets and subsequently transferred to a spinning bioreactor for enhanced nutritional absorption, enabling the organoids to grow up to a few millimeters in diameter. Unlike the Eiraku et al. method [32], the Lancaster approach does not employ region-specific patterning factors to guide progenitors to particular regional identities. Instead, the use of an extracellular scaffolding matrix enables the growth and extension of neuroepithelium buds that will eventually self-organize and develop into various brain regions. Importantly, the use of Matrigel also introduces undefined animal factors into the culture that often leads to variations in organoid size and morphology and to inconsistent generation of brain regions in each organoid, thus limiting their potential to accurately model many aspects of neurodevelopment and degenerative diseases. Another source of inconsistency comes from the hPSC lines themselves, due to donor or manufacturing-induced variability that could impact reproducibility of cerebral organoids. Nevertheless, a subsequent study compared cell fate and regional specificities in cerebral organoids using single-cell RNA sequencing and showed that the organoid cortical cells followed a gene expression program that closely mimicked that of the human fetal neocortex [35]. Thus, the 3D cerebral organoid model may be useful to explore the genetic underpinnings of human corticogenesis.

To reduce cost and space requirements of the Lancaster et al. spinning reactor approach, Qian et al. developed a miniature spinning bioreactor, named SpinΩ, that fits in a 12-well tissue culture plate and allows for larger scale generation of cerebral organoids [36]. Furthermore, unlike the Eiraku and Lancaster approaches [32, 34], Qian et al. pre-patterned EBs formed from intact hPSC colonies using brain region-specific patterning factors for 7 days. The EBs were then embedded in Matrigel for another 7 days, removed from the Matrigel, and further cultured in SpinΩ. This approach enhanced organoid culture reproducibility and successfully generated different brain region-specific organoids, including forebrain, midbrain and hypothalamic organoids.

Paşca et al.*,* on the other hand, successfully demonstrated the shift from neurogenesis to gliogenesis in hPSC-based 3D culture using a novel organoid model named human cortical spheroids (hCSs) [37]. The hCS protocol begins with differentiating intact suspended hPSC colonies into the folded spherical structures using BMP and TGF-β signaling inhibitors, dorsomorphin and SB-431542, respectively. The floating spheroids are exposed to bFGF and epidermal growth factor (EGF) for 19 days to prime neural progenitors for the generation of nonreactive astrocytes, which appear after the peak of neurogenesis [38, 39]. Neurons in hCSs are surrounded by nonreactive astrocytes which facilitate formation of functional synapses [37]. Furthermore, hCSs are made up of neurons from deep and superficial cortical layers and their transcriptional signatures mimic those found during in vivo fetal development. Therefore, hCSs provide a new avenue to model the patterning and specification of various neuronal and glial cell types.

**Fig. 1:**
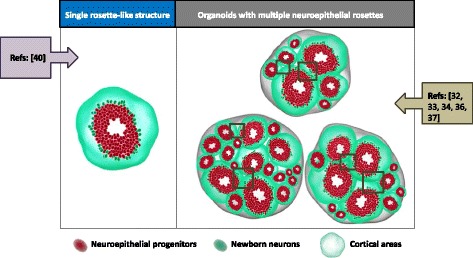
hPSC-based 3D brain organoid models with single or multiple, varying sizes and numbers of ventricular zone (VZ)-like regions. Examples of hPSC-derived 3D brain organoid models are divided into models that contain single rosette-like structure (left panel) or randomized sizes and numbers of neuroepithelial rosettes (*right panel*). Overlapping regions (*box*) interrupt the cortical plate layer formation and create difficulties with reproducibility and quantification of the cytoarchitecture of brain organoids

Besides the great potential of these 3D models, there are still several limitations inherent in these hPSC-based organoid culture systems. First, these brain organoids contain multiple and variable sizes and numbers of ventricular zone (VZ)-like regions which create difficulties with reproducibility and quantification of the cytoarchitecture (Fig. 1). Secondly, randomized generation of neuroepithelial rosettes in the case of cerebral organoids could interrupt cortical plate layer formation between borders of adjacent cortical structures, further affecting reproducibility (Fig. 1). Recently, Lee et al. established a hPSC-based 3D neocortical organoid model that stemmed from a single rosette-like structure (Fig. 1), using defined patterning molecules and neocortical trophic factors without extracellular scaffolding [40]. They indicated that the size of hPSC-derived neuroepithelial rosettes, which are manually dissected for use in subsequent neocortical organoid differentiation, is critical for achieving sufficient neocortical organoid development, with small rosettes being unable to successfully differentiate. This 3D neocortical organoid approach employs dual SMAD and FGF signaling inhibition during EB formation. The floating EBs are then grown in adherent culture to generate appropriate size of rosettes with dorsopallial identity, ranging from 50,000 to 200,000 μm^2^ in size, manually dissected, and maintained in suspension culture with bFGF and FGF18. The resulting organoids are cultured in individual wells of 96-well plates without any trophic factors to enable self-organized neocortical organoid formation. They retain essential features of neocortical development, including a proliferative neuroepithelium at earlier development stages, neurogenesis, neuronal migration, and later enlargement of the neocortical area. Moreover, this 3D hPSC-based neocortical organoid model stemming from a single neocortical unit without scaffolding support substantially enhances reproducibility of brain organoid generation, and has potential to develop into an innovative platform for pharmacological applications requiring quantification.

## Modeling developmental disorders with 3D brain organoids

3D iPSC-derived brain organoids from patients with neurodevelopmental disorders can recapitulate pathological phenotype in a dish. In addition, hPSC-derived 3D brain organoids enabled in vitro studies of cytoarchitectural changes in the embryonic neocortex as a result of perinatal teratogen exposure such as viruses and drugs. In this section, we summarize recent findings using hPSC-based 3D brain organoids for neurodevelopmental disease modeling and therapy development strategies.

### Microcephaly

Autosomal recessive primary microcephaly (MCPH) is a neurodevelopmental disorder characterized by smaller brain, particularly affecting cerebral cortex size [41, 42]. Twelve genes have been implicated in MCPH and the majority of them encode centrosomal proteins that play a role in mitotic progression [41]. Among these MCPH genes, CDK5RAP2 regulates centriole replication and loss of *CDK5RAP2* has been shown to impact proliferation of neural progenitors [43, 44]. Nevertheless, *CDK5RAP2* mutant mice did not exhibit a severely reduced brain size as was observed in human patients [43, 45]. Lancaster et al. used iPSCs derived from a microcephalic patient with a heterozygous nonsense mutation in *CDK5RAP2* to model the progression of microcephaly in 3D cerebral organoids [34]. Compared to control, patient-derived organoids displayed smaller neuroepithelial regions, altered spindle orientation of radial glial cells, and abundant neuronal outgrowth. Importantly, patient-derived cerebral organoids overall were smaller in size compared to controls, similarly to the reduced brain size seen in patients. Their findings further indicated that smaller organoid size is a consequence of impaired proliferation and expansion of the founder progenitor pool and simultaneous premature neuronal differentiation. Their data also suggest that mouse neural progenitors prior to neurogenesis do not proliferate and expand to the same degree as in humans which might explain why *CDK5RAP2*-deficient mice do not exhibit microcephaly with the same severity as humans [46, 47].

### Zika virus and microcephaly

Recently, the Zika virus (ZIKV) outbreak and the causal relationship between ZIKV infection during pregnancy and microcephaly in newborns had spurred the World Health Organization (WHO) to declare a global public health emergency [48–51]. ZIKV has been identified in the placenta, amniotic fluid, blood, and brains of microcephalic fetuses [49, 50, 52–55]. Nevertheless, the impact and mechanisms underlying the adverse neurodevelopmental effects of ZIKV are still largely unknown. Recently, ZIKV was shown to infect hiPSC-derived neural progenitors in 2D culture, and infected human neural progenitors were found to release infectious ZIKV particles [56]. Moreover, several recent studies using 3D hPSC-derived cerebral organoids showed that ZIKV infection causes an overall reduction in organoid size, including a significant decrease in both ventricular zone (VZ) and cortical plate thickness [36, 57–59]. They also demonstrated that ZIKV induced cell death in neuroepithelial progenitors, attenuated the proliferation of progenitor cells, and increased the size of lumen within ventricular structures. These results are consistent with a clinical case report describing enlarged ventricles observed in a ZIKV-infected human fetal brain [60]. Dang et al. indicated that Toll-like-Receptor 3 (TLR3), the innate immune receptor, was upregulated in hPSC-derived cerebral organoids infected with ZIKV, and blockade of TLR3 reduced ZIKV-induced organoid shrinkage and apoptosis [57]. Transcriptomic analysis further revealed potential genes regulated by TLR3, including *NTN1* and *EPHB2* by which ZIKV may cause apoptosis and impact neurogenesis in the developing cerebral organoids. Intriguingly, both ZIKV and dengue virus, members of the flavivirus genus, have been shown to lead to activation of innate immune responses and increase the expression of *TLR3* [61, 62]; however, only ZIKV would cause microcephaly [63, 64] and induce the adverse effects observed in 3D organoid culture [58]. Therefore, further studies will be required in order to clarify a causal relationship between TLR3-mediated innate immune response and ZIKV-induced microcephaly.

### Perinatal drug exposure and brain development

Prenatal exposure to many illicit drugs, alcohol, and tobacco has the potential to impact fetal brain development and continues to be a significant public health problem [65–68]. Of these substances, cocaine exposure during fetal development is most consistently linked to impairments of fetal head growth and subsequent neurobehavioral defects [67, 69–73]. Notably, it has been suggested that prenatal cocaine exposure is associated with impaired neurobehavioral function through disruption of frontal cortical development [74–76]. Lee et al. demonstrated that prenatal cocaine exposure during the most active period of neural progenitor proliferation induced deleterious cytoarchitectural changes in the embryonic neocortex in rats [77]. These cytoarchitectural changes are initiated by N-oxidative metabolism of cocaine and consequent oxidative ER stress signaling [78]. However, because of human and rodent interspecies differences in neocorticogenesis [79, 80] and CYP-mediated drug metabolism [81], findings from rodent models have been difficult to extrapolate to human brain development. Using a 2D hPSC-based neocortical model, Kindberg et al. demonstrated that cocaine caused premature neuronal differentiation of various cortical neuronal subtypes and impaired neocortical patterning [23]. Using hPSC-based 3D neocortical organoids with a single cortex-like unit, they further identified a specific human cytochrome P450 isoform, CYP3A5, to be responsible for cocaine-induced developmental abnormalities of the human neocortex, which include both proliferation deficit and premature differentiation of neuroepithelial progenitors along with a significant reduction in cortical plate formation in organoids [40]. 3D organoid methodology may therefore provide an alternative approach to study adverse effects of abused psychostimulants.

### Autism spectrum disorder

Autism spectrum disorder (ASD) is a complex disorder of brain development characterized by language deficits, social communication difficulties, and repetitive behaviors. MRI scans and post-mortem studies of ASD patients indicate abnormal brain development owing to initial brain overgrowth followed by premature growth arrest, and these alterations are most evident in the prefrontal cortex [82–85]. Alterations in cerebral development in ASD lead to disorganization of cortical laminar architecture and changes in cortical networks that may result in autistic behaviors.

The majority of cases of ASD are idiopathic, therefore the exact genetic causes of autism are not known. Recently, Mariani et al. directly modeled early ASD brain development using iPSC-derived cerebral organoids from idiopathic ASD patients with common macrocephalic phenotype [86]. ASD-derived organoids exhibited a decrease in cell cycle length in neural progenitor cells during the early stages of organoid differentiation, enhanced synaptic maturation, and overproduction of GABAergic inhibitory neurons. Their findings further demonstrated that FOXG1 and its downstream genes were responsible for the phenotypic abnormalities identified in ASD-derived organoids. Using a 2D neural differentiation model, another study with macrocephaly ASD revealed enhanced proliferation of neural progenitor cells resulting from dysregulation of WNT transcriptional cascade, and defects in the neuronal networks that could be reversed by insulin growth factor 1 (IGF-1), a drug currently in clinical trials for ASD [87].

Although the exact genetic causes of ASD are unknown, it is thought that several complex genetic factors might be involved. Copy-number variations (CNVs) are the most common recognized genetic variations associated with autism, and approximately 10–20% of autism subjects exhibit at least one CNV [15, 88, 89]. Yet, the impact of each particular CNV on brain development or function in autism is not known. A number of studies have linked CNVs to autism, including several CNVs on chromosome 17 at or near 17q21.31–21.32. Notably, one autistic subject was identified with a de novo mutation comprising of a duplication of 17q21.31-17q21.32/*WNT3*-*WNT9B* [90]. Lee et al. recently reported that hPSC lines with a duplication of 17q21.31-17q21.32, including *WNT3* and *WNT9B*, exhibited enhanced proliferation of early hPSC-derived neural progenitor cells, and increased neuronal differentiation at later culture stages [5]. Their findings support Marchetto et al.*’s* assumption that WNT signaling is implicated in the pathogenesis of autism [87]. These findings demonstrate the potential of using hiPSC technology to study aberrant neurodevelopmental processes in complex human diseases exhibiting heterogeneity in genotypes, such as ASD. Advancements in 3D cerebral organoid systems may provide an innovative platform for finding effective treatments for autism, potentially targeting WNT pathways.

## Modeling neurodegenerative disorders with 3D brain organoids

Most neurodegenerative disorders first appear in adulthood. To recapitulate late-onset diseases, the fetal nature of hPSC-derived neural cells must be taken into consideration. In some cases, earlier-onset neurologic disease variants such as early-onset AD are available for hiPSC-based disease modeling [91, 92]. Recently, using early-onset familial Alzheimer’s (fAD) patient-derived iPSCs with *APP* duplication, Raja et al. recapitulated Alzheimer’s disease phenotypes including β-amyloid (Aβ) aggregation, hyperphosphorylated Tau (pTau), and endosome abnormalities in fAD patient-derived 3D brain organoids [93]. Moreover, both amyloid and pTau pathologies in fAD organoids were reduced by treatment with β- and γ-secretase inhibitors. Therefore, 3D brain organoids may be a useful tool for drug screening for neurodegenerative disorders.

Nevertheless, early-onset fAD is a rare form of AD, accounting for less than 5% of all cases [91, 92]. In order to model late-onset neurologic diseases, the immature features of hPSC-derived brain organoids must be overcome. Long-term differentiation is a rational approach for further development of mature brain organoids, yet lack of vascularization seems to limit their prolonged growth potential. Vascularization does not participate in early development of the neocortex but is involved in regulating neurogenesis and guiding neuronal migration into the neocortex during the late stages of cortical development [94–96]. Moreover, impaired passage of nutrient and oxygen deep within organoids leads to necrosis at the center and interferes with organoid maturation. Absence of immune cells is another limitation and restricts the use of brain organoids in modeling inflammatory responses to infection or toxic substances, and aging-associated inflammation. It is worth noting that, due to the lack of vasculature and immune cells, the ability of hPSC-based 3D brain organoid model to mimic both young and aged brains remains controversial.

## Potential improvements of hPSC-based 3D brain organoid model

The structural complexity of the human brain makes it difficult to detect all pathological conditions of neurological disorders in vitro. Recruitment of vascular networks, immune cells, or even blood-brain barrier within organoids would be beneficial for their advanced growth and differentiation and make them a more physiologically relevant model of the human brain. Recently, Takebe et al. demonstrated that transplantation of organ buds generated using brain tissues with mesenchymal stem cells facilitated organoid vascularization [97]. Moreover, microglia, the only resident immune cells of the CNS, are suggested to regulate synaptic pruning and thus contribute to the development and maintenance of neural circuits; they have also been linked to early progression of neurodegenerative disease [98–103]. Microglia are absent from hPSC-derived brain organoids due to an embryonic origin distinct from that of neural progenitors. Microglia are derived from primitive myeloid progenitors in the embryonic yolk sac, and invade the brain during embryonic development [104–106]. Muffat et al. and Pandya et al. established protocols for efficient generation of microglia from hPSCs, which suggests a possibility for microglia to be incorporated into brain organoid cultures during early stages of organoid development [107, 108]. Recently, Schwartz et al. combined hPSC-derived neural progenitors, endothelial cells, mesenchymal stem cells, and microglia precursors on chemically defined polyethylene glycol hydrogels to establish 3D neural constructs with microglia and vascular network [109]. These findings emphasize the importance of developing vascularized brain organoids and support the feasibility of introducing microglia into developing brain organoids to enhance neuronal maturation within these organoids.

## Therapeutic development strategies of neurological disorders using 3D brain organoid technology

The successful use of iPSC-derived 3D organoids for disease modeling, specifically for neurological disorders (Table 2), suggests a potential for 3D organoids in drug screening and development of innovative diagnostic and therapeutic strategies. While animal models are still widely used for drug screenings and therapeutic testing, there are several limitations associated with them, including the lack a reliable animal model for many neurological disorders [110] and the financial/logistical difficulties of small-molecule high-throughput screening (HTS) using animal models [111–113]. The iPSC-derived 3D organoid model offers a practical and less expensive alternative for drug screening, with the added benefit of patient-specific, genetically-relevant drug efficacy and toxicity data obtainable using iPSCs.

**Table 2:** Modeling neurological disorders with 3D brain organoids derived from human pluripotent stem cells

Disease	Gene/Substance	Type of PSCs	Brain region	Disease phenotype in organoid	Disease mechanism	Therapeutic strategies	Reference
Microcephaly	*CDK5RAP2*	Human iPSCs	Cerebral cortex	Smaller neuroepithelial regions, altered spindle orientation of radial glial cells, abundant neuronal outgrowth, smaller organoid size	Heterozygous nonsense mutation in *CDK5RAP2*	Reintroducing CDK5RAP2 protein	[34]
Impaired brain growth	ZIKV	Human iPSCs	Forebrain	Increased cell death and suppressed proliferation of neural progenitors, decreased neuronal layer thickness and organoid size, enlarged lumen/ventricles	-	-	[36]
Impaired brain growth	ZIKV	Human ESCs	Cerebral cortex	Attenuated brain organoid growth	TLR3-mediated dysregulation of neurogenesis	TLR3 inhibitor	[57]
Impaired brain growth	ZIKV	Human iPSCs	Cerebral cortex	Reduced viability and growth of neural progenitor cells, smaller brain organoid size	-	-	[58]
Impaired brain growth	ZIKV	Human ESCs/iPSCs	Cerebral cortex	Increased apoptosis in neural progenitors, reduction of prolifration zone, disrupted cortical layers	-	-	[59]
Impaired brain growth	Cocaine	Human ESCs	Neocortex	Proliferation inhibition of neuroepithelial progenitors, premature neuronal differentiation, reduction in cortical plate formation	CYP3A5-mediated cocaine oxidative metabolism	CYP3A5 inhibitor/Knockdown of CYP3A5	[40]
Autism spectrum disorder/macrocephalic phenotype	-	Human iPSCs	Dorsal telencephalon	Increased progenitor cell proliferation, enhanced synaptic maturation, overproduction of GABAergic inhibitory neurons	Overexpression of transcription factor *FOXG1*	Knockdown of FOXG1	[86]
Early-onset familial Alzheimer’s	*APP*	Human iPSCs	Neocortex	β-amyloid (Aβ) aggregation, hyperphosphorylated Tau (pTau), endosome abnormalities	*APP* duplication	β- and γ-secretase inhibitors	[93]

**Fig. 2:**
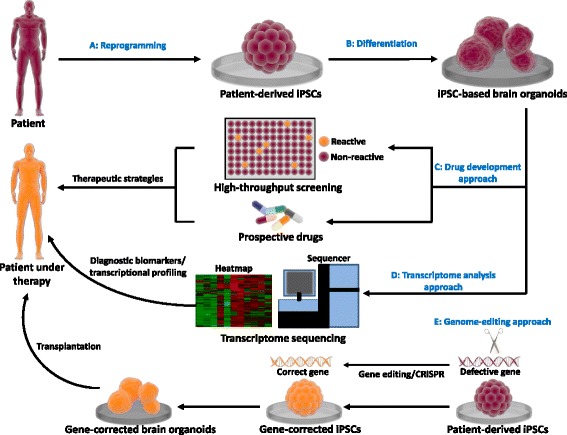
Therapeutic development strategies using hiPSC-based 3D brain organoid technology. Patient-derived iPSCs can be differentiated into brain organoids exhibiting disease-specific phenotypes (**a**,**b**). Three main approaches can be used to develop innovative therapeutic strategies (C-E). (**c**) For drug development strategies, HTS enables a large number of chemicals to be screened, but the prospective drug approach only allows a small number of defined drugs to be examined on brain organoids. (**d**) For transcriptome analysis, RNA sequencing (RNA-Seq) along with brain organoids provides an opportunity for scientists in studying the transcriptional profiling of the human complex neurological disorders. (**e**) For genome-editing approach, CRISPR-Cas9 can correct a genetic defect associated with disease phenotypes in brain organoids. Gene-corrected brain organoids could be used to replace impaired brain tissue via transplantation

Brain organoids generated from patient-derived iPSCs can be used to model brain development and neurodegenerative disorders (Fig. 2a, b). Once the disease-specific phenotypes are identified in patient-derived brain organoids, there are three potential approaches to develop novel therapeutic strategies (Fig. 2c-e). In the drug development approach (Fig. 2c), HTS enables a large number of drug-like compounds to be tested on brain organoids with phenotypes that can be automatically evaluated. HTS is advantageous for developing novel treatments in neurological disorders. In contrast to HTS, the prospective drug approach, which examines effects of a small number of defined drugs for attenuation of neuropathic phenotypes in organoids, is valuable while the mechanisms involved in neurological disorders are known. In transcriptome analysis approach (Fig. 2d), genome-wide expression analysis in patient-derived brain organoids using RNA sequencing (RNA-Seq) provides an opportunity for in-depth transcript profiling of fundamental molecular mechanisms involved in the pathogenesis of complex neurological diseases. Recent studies have been rather encouraging that transcriptome sequencing of organoids may help to identify novel diagnostic biomarkers and enable more personalized treatment regime [114–116]. Development of pharmacotherapies that interrupt or reverse gene expression changes would be beneficial to the treatment strategies. In genome-editing approach (Fig. 2e), patient-derived organoids harboring genetic defects can be employed to define the role of mutated genes that are suspected to cause the disease using genome-editing technologies such as CRISPR-Cas9 [117, 118]. In addition, repaired patient-derived organoids using genome-editing techniques could be a potential option for replacing impaired brain tissue via transplantation [119–121].

## Conclusion

In this review, we described past milestones and present state of the field of brain organoid research, paying particular attention to the ability of current models to recapitulate brain development and neurodegenerative disorders. Although our understanding of the self-organizing properties of brain organoids is rapidly advancing, the generation of individual, discrete brain regions remains a challenge. The absence of immune and vascular systems in cultured brain organoids not only impedes their growth and maturation but also limits their use in modeling certain neurological disorders. However, these deficiencies might be improved by alternative strategies applying newly developed systems, such as vascularized organ buds, microglia generated from hPSCs, or the incorporation of vascular and microglial components into neural constructs [97, 107–109]. In addition, microfluidic networks can be employed to deliver patterned signals to brain organoids to further enhance their development and maturation [119]. Despite hurdles to be overcome, these near-physiological brain organoid models represent orthogonal approaches that have been able to recapitulate characteristics of various neurological disorders in a self-organized 3D human neural tissue. In combination with transcriptome profiling, brain organoids can be employed to study functional effects of the dynamic expression of genetic risk factors in neurodevelopmental and neurodegenerative diseases. Advancements in iPSC-derived brain organoids that authentically recapitulates human brain development could potentially make 3D brain organoid culture systems an innovative platform for developing effective treatments for neurological disorders, including the use of HTS assays to identify small molecules that target neurological diseases, transcriptome sequencing to identify diagnostic biomarkers and uncover the molecular mechanisms, and the use of genome editing of hiPSCs for personalized cell replacement therapy. Although much remains to be done, recent studies and results support the potential of iPSC-derived 3D brain organoids in modeling and facilitating treatment of neurological disorders.
